# Severe haemophilia A in a preterm girl with Turner syndrome: case report – a diagnostic and therapeutic challenge for a paediatrician (Part 2)

**DOI:** 10.1186/s13052-021-01103-7

**Published:** 2021-07-13

**Authors:** Berendt Agnieszka, Wójtowicz-Marzec Monika, Wysokińska Barbara, Kwaśniewska Anna

**Affiliations:** 1 Department of Obstetric and Pathology of Pregnancy, Medical University of Lublin, Staszica 16, 20-081 Lublin, Poland; 2 Department of Pediatric Cardiology, Medical University of Lublin, Prof. A. Gębali 6, 20-093 Lublin, Poland

**Keywords:** Haemophilia, Turner syndrome, Infant premature, Infant newborn, Genetic diseases, inborn, Blood coagulation disorders, inherited, Gonadal dysgenesis, vaccination, Factor VIII, Factor VIII/adverse effects

## Abstract

**Background:**

Haemophilia A is an X-linked genetic condition which manifests itself mainly in male children in the first 2 years of life, during gross motor skill development. This disorder is rare in females. The clinical manifestation of severe haemophilia in preterm infants poses a great challenge to the therapeutic team. As extreme prematurity is linked to an increased risk of central nervous system or gastrointestinal bleeding, a well-informed and balanced treatment from the first days of life is crucial to prevent long-term damage.

Haemophilia is most commonly caused by inheriting defective genes, and can also be linked to skewed X inactivation and Turner syndrome.

The coincidental occurrence of haemophilia A and Turner syndrome is extremely rare, with only isolated cases described to date. Hence, a multidisciplinary approach is needed.

**Case presentation:**

The authors report on a preterm girl (gestational age 28 weeks) diagnosed with haemophilia and Turner syndrome. The first manifestation of haemophilia was prolonged bleeding from injection sites on the second day of life. Indeterminate aPTT and factor VIII level < 1% confirmed the diagnosis of haemophilia A. Dysmorphic features which did not match the typical clinical picture of haemophilia, the female sex, and a negative paternal family history led to the diagnosis of Turner syndrome. While in hospital, the girl received multiple doses of recombinant factor VIII in response to prolonged bleedings from the injection sites and from a nodule on the girl’s head, and before and after retinal laser photocoagulation. No central nervous system or abdominal cavity bleeding was observed. The substitutive therapy was complicated by the development of factor VIII inhibitor (anti-factor VIII (FVIII) antibodies). Treatment was continued with recombinant factor VIIa. This article aims at demonstrating the complexity of the diagnostics and treatment of a preterm child with two genetic disorders.

**Conclusions:**

Haemophilia should always be considered in the differential diagnosis of prolonged bleeding, even in patients with a negative family history. In the case of coinciding atypical phenotypic features, further diagnostics for another genetic disease are recommended. Infant care should follow current care standards, while considering certain individual features.

## Background

Haemophilia A, caused by factor VIII deficiency, is an X-linked recessive genetic disorder which leads to impaired blood coagulation functions. It is considered a rare disease. The prevalence of haemophilia A is 1–9/100,000 [1].

According to the database of the I*nstitute of Haematology and Transfusion Medicine in Warsaw, the number of* haemophilia A patients in Poland in 2018 was 2253. In 148 patients, an inhibitor to a given clotting factor was found [2].

Haemophilia A is a X-linked recessive condition. Two copies of the haemophilia gene must be inherited to be affected that is why the disease is very rare in women. A female haemophilia patient inherits a defective gene on the X chromosome from both her mother (a carrier) and her father (a sufferer). Women are usually carriers of the defective gene. Haemophilia is most frequently found in men who have only one X chromosome, which they inherit from their disease-carrier mothers [3].

When a father is healthy, haemophilia might manifest in females due to skewed X-inactivation or monosomy X [4, 5].Skewed X inactivation is when 80% or more of the cells show a preferential inactivation of one X chromosome [6]. If a genetic female haemophilia carrier has extreme X chromosome inactivation, phenotypically she suffers from haemophilia.

The discussed case presents the third possibility for haemophilia A occurrence in females, namely its coincidence with Turner syndrome. Turner syndrome (45X) has genetic origins, but it is not hereditary. It is connected with the complete or partial monosomy of an X chromosome [4]. Its prevalence is reported as 1 in 2500 female births; however, it is estimated that the actual number is higher [4]. Most pregnancies with Turner syndrome result in spontaneous miscarriage. Moreover, some cases whose clinical presentation is atypical are diagnosed as late as in adolescence or even adulthood.

The spontaneous mutations which occur within the gene for haemophilia can also induce symptoms [3].

**Fig. 1:**
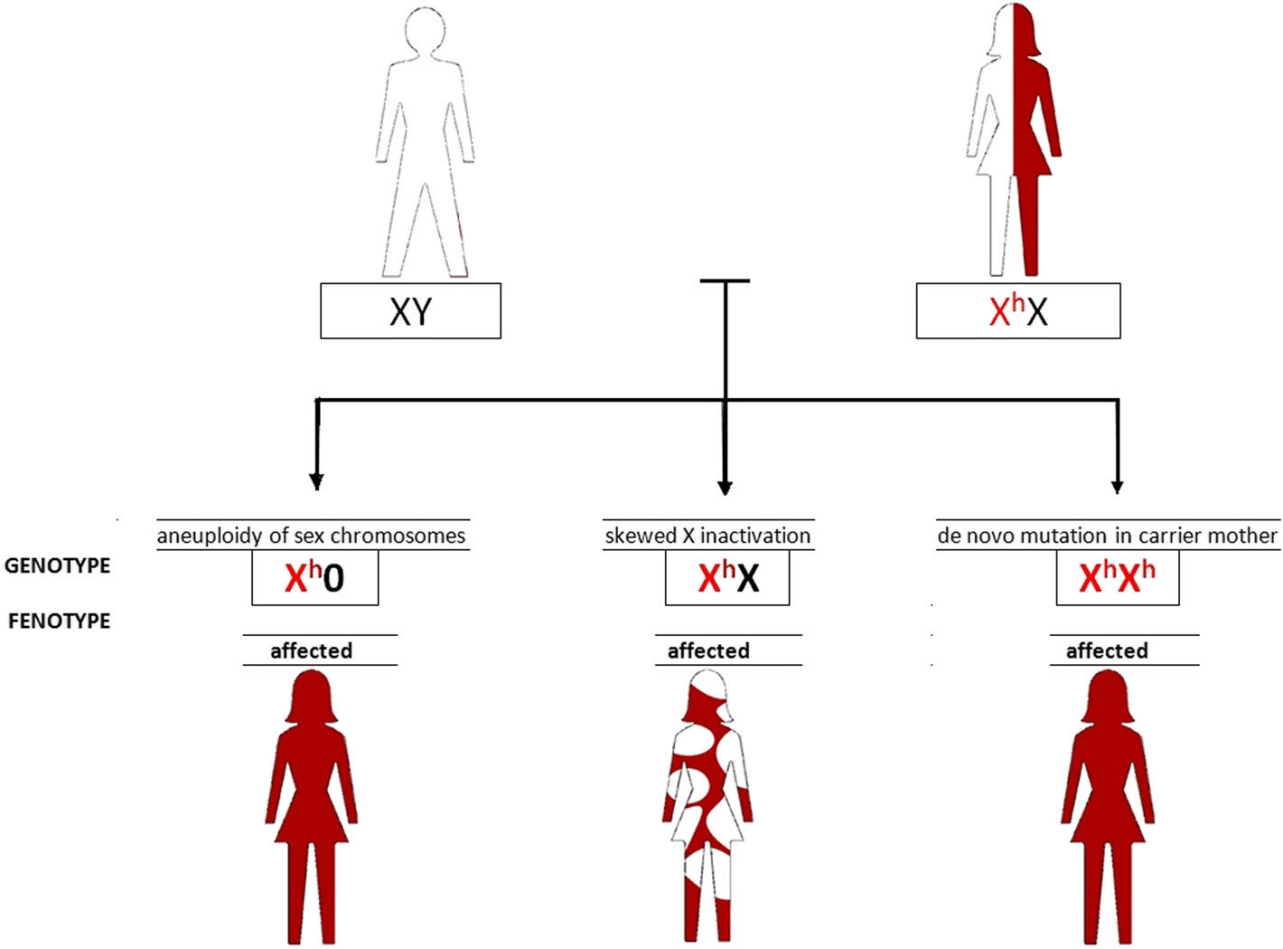
Scheme of possible causes of hemophilia in girls of healthy father and carrier mother (personal authorship)

Figure 1 presents possible causes of haemophilia disease in girls of healthy father and carrier mother.

The reported case involves a girl with features of extreme immaturity and an early manifestation of severe haemophilia A, who also was diagnosed with Turner syndrome. The coincidence of these disorders is very rare [7]. The available literature in the PubMed database cites 8 cases (this article describes the ninth) of haemophilia and Turner syndrome coincidence [7]. The presented case report is the first case of such concurrent diagnosis in a preterm child [7].

The aim of this article is to raise awareness of the incidence of haemophilia in female infants. It is worth emphasising that a proper diagnosis of this disorder requires a thorough patient history and physical examination.

The article also draws attention to the differences of therapeutic and preventive managements in the care of preterm infants with haemophilia.

## Case presentation

A female preterm infant (second pregnancy, second labour) was born at 28 weeks gestation, through Caesarean section, due to the risk of perinatal asphyxia and vaginal bleeding in the mother.

The first pregnancy was resolved on the due date through a C-section because of foetal macrosomia. In the postpartum period the mother experienced heavy bleeding from the birth canal. She had a positive family history – the mother’s brother suffered from severe haemophilia A, diagnosed at the age of 3. According to her mother, there had been no previous episodes of postoperative or postpartum bleeding in the family. The child’s father was healthy.

The preterm infant was small for gestational age (SGA) (body weight: 880 g). After birth, the girl was administered vitamin K as a preventive treatment for neonatal haemorrhagic diathesis. The procedures after birth included mechanical ventilation, the administration of a surfactant, parenteral feeding, and diagnostics for congenital infection.

A physical examination revealed significant features of extreme immaturity. Moreover, certain features of dysmorphia in the infant were found (big low-set ears, micrognathism, gothic palate, widely spaced nipples single transverse palmar crease) [7], suggesting a karyotypic abnormality. At the 36th hour of life, the girl had an episode of bleeding from the injection sites. The bleeding did not stop after the re-administration of vitamin K and cyclonamine, and was managed only after the girl was given fresh frozen plasma (FFP). The coagulation times monitored on the second day of the girl’s life revealed an indeterminate aPTT and normal PT. Coagulation factors performed on the sixth day of the child’s life (i.e. 4 days after FFP administration) showed a low level of factor VIII (1%) and normal levels of factors IX, XI, XII, and the von Willebrand factor. A circulating anticoagulant test yielded a negative result. A control test of factor VIII on the tenth day of the infant’s life yielded the result of 0.1%, suggesting severe haemophilia A (Table 1).

**Table 1:** Results of coagulation tests during hospitalization

Day of life	PT (11–14 s.) [8]	INR (0,8-1,3) [8]	APTT (31–65 s) [9]	Factor VIII (65,2-153,4%) [10]	Factor IX (30–77%) [11]	Factor XI (32,9–75%) [12]	Factor XII (25–81%) [13]	vWF (46–219,5%) [14]	vWF activity (73,7-188,9%) [15]	anti-Factor VIIIantibodies (Bethsa units)
2	15.4	1.24	indeterminable							
6	14,1	1,14	85	1	34,8	33,7	19,7	248,3	351	no
10	14,4	1,17	102,1	0,1						
75			indeterminable	0,1						9,1
85			indeterminable	0						25

*PT* prothrombin time, *APTT* Activated Partial Thromboplastin Time, *INR* international normalized ratio, *vWF* von Willebrand factor

**Fig. 2:**
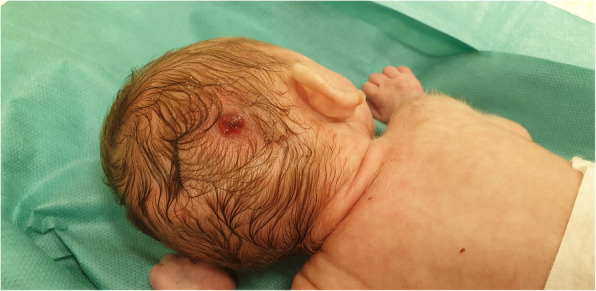
The patient at 24 day of life A nodule with oozing blood on the head. The bleeding stopped after the administration of factor VIII (written consent to publish was obtained from the patient’s parents)

In the course of hospitalisation, in the third week of the girl’s life, a nodule appeared on her head, which was oozing blood (Fig. 2). The bleeding stopped after the administration of factor VIII.

Moreover, during hospitalisation the infant received multiple doses of recombinant factor VIII in response to prolonged bleedings from the injection sites and as a preventive measure before a retinal laser photocoagulation procedure, which was performed due to the stage 2 retinopathy of prematurity with plus disease. Following the photocoagulation procedure, a subretinal haematoma was found, which required subsequent doses of factor VIII over the next 3 days, which led to the gradual receding of lesions in the fundus of the eye.

**Fig. 3:**
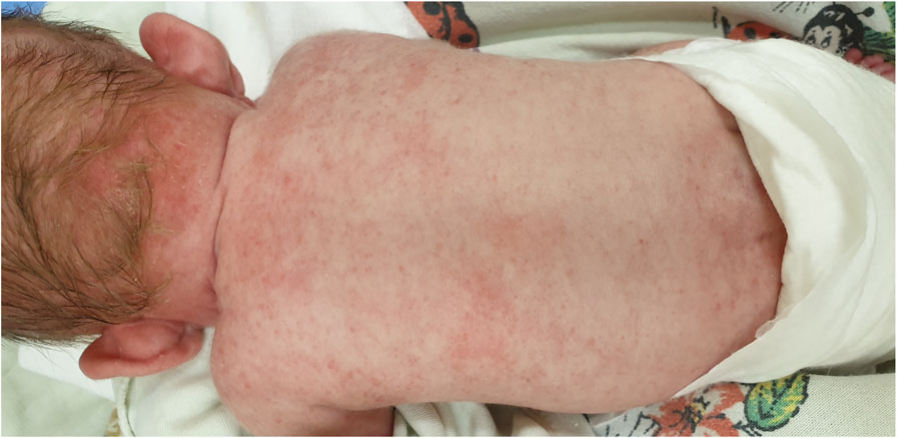
The girl in 53 day of life. Rash after VIII factor infusion. Rash dissappeared within following week (written consent to publish was obtained from the patient’s parents)

The girl required the administration of several doses of packed red blood cells. An allergic rash was observed after the second supplementary transfusion of packed red blood cells (Fig. 3).

**Fig. 4:**
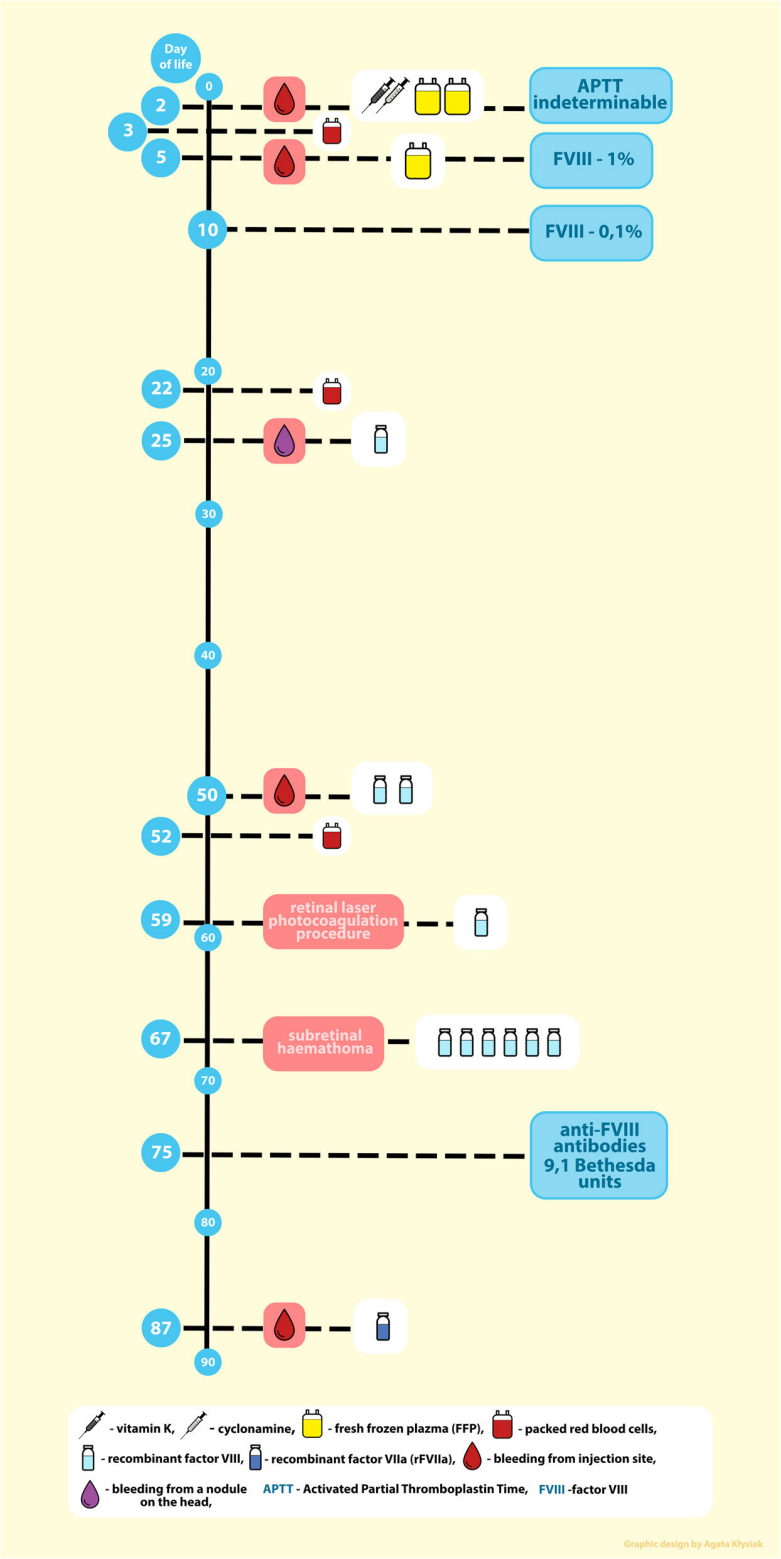
The most relevant events of the patient medical history and their management

On the 75th day of the girl’s life, following a total number of 9 factor VIII doses, the factor VIII inhibitor level (anti-Factor VIII (FVIII) antibodies level) was determined at 9.1 Bethesda units. The control tests of the inhibitor level, showed a rising trend for the inhibitor titre (Table 1). Any subsequent bleeding episodes were treated with recombinant factor VIIa (rFVIIa). The most relevant events of the patient medical history and their management are presented in the Fig. 4.

**Fig. 5:**
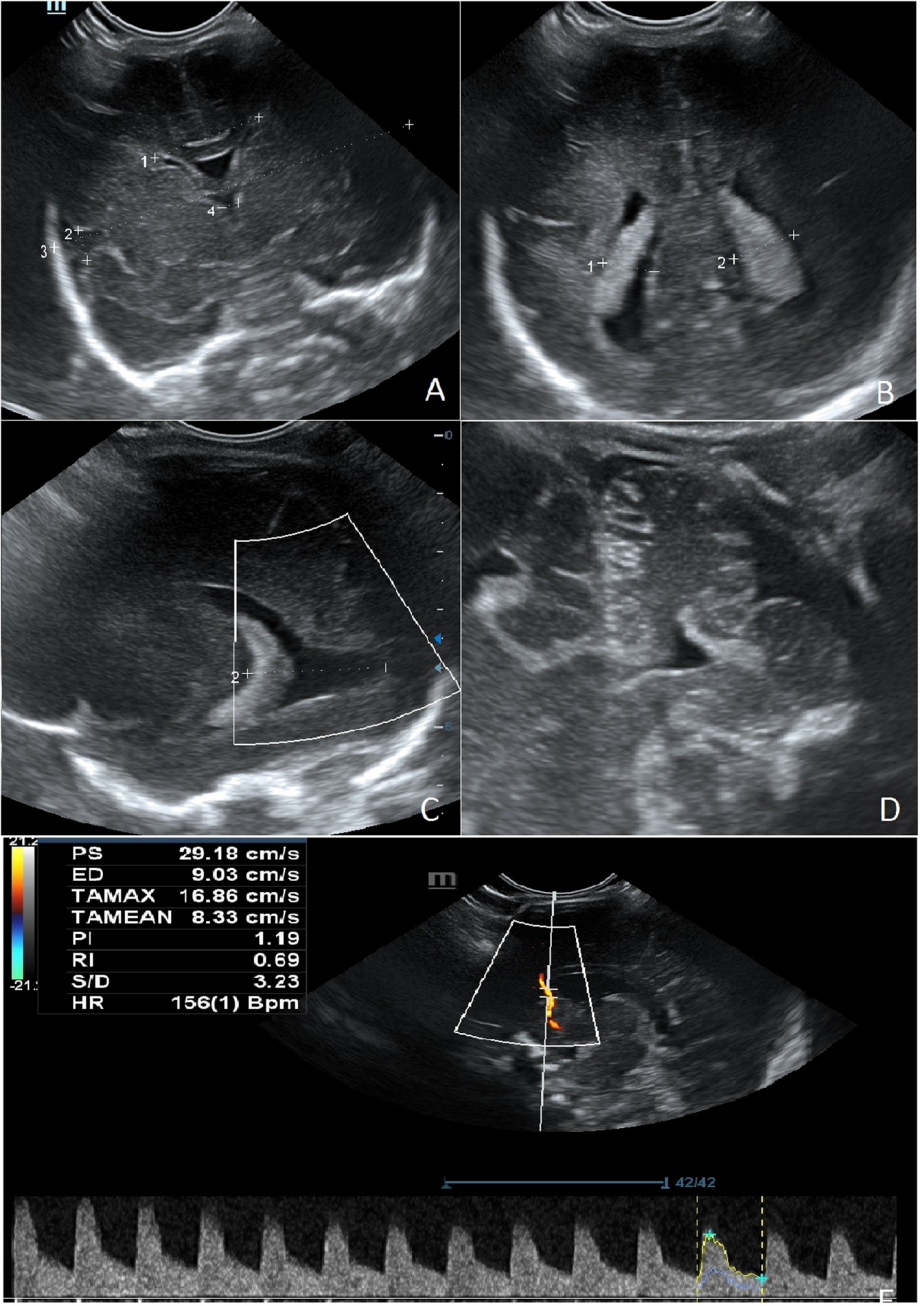
Cranial sonography at the age of 30 weeks corrected age. **A** Coronal scan showing normal frontal horns, cavum sepum pellucidi, third ventricle. **B** Choroid plexus in lateral ventricles in coronal scan. **C** Scan of normal occipital horn. **D** Scan of normal cerebellum visualized by mastoid window, **E** Normal Doppler parameters of blood flow in the arteria cerebri anterior. (written consent to publish was obtained from the patient’s parents)

No bleeding to the central nervous system was observed (Fig. 5). Vaccines were administered subcutaneously in accordance with the applicable immunisation schedule. No bleeding was observed during subcutaneous vaccinations. No RSV prevention (Palivizumab) was given due to the necessity of its intramuscular administration.

**Fig. 6:**
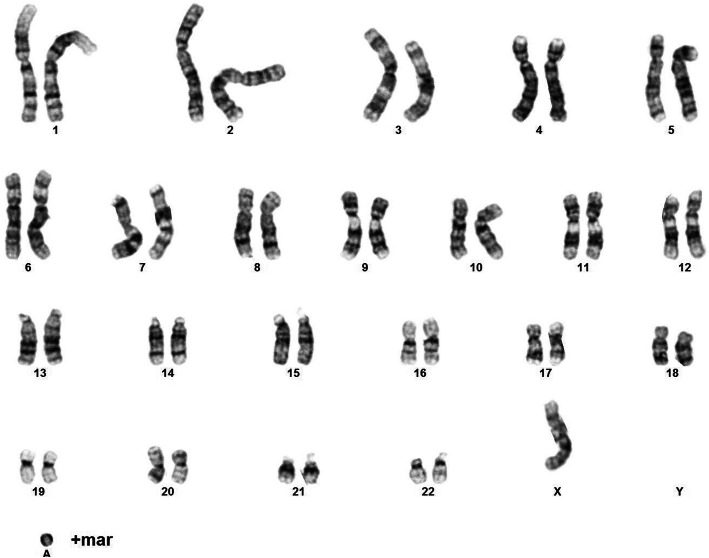
Patient’s karyotype 46,X,+mar [26]/45,X [4] (written consent to publish was obtained from the patient’s parents)

The haemophilia diagnosis, dysmorphic features, negative paternal history were indications for karyotyping. It revealed the presence of two cell lines, 45,X/46,X,+mar, which confirmed the diagnosis of Turner syndrome (Fig. 6). The presence of the SRY gene was ruled out.

**Fig. 7:**
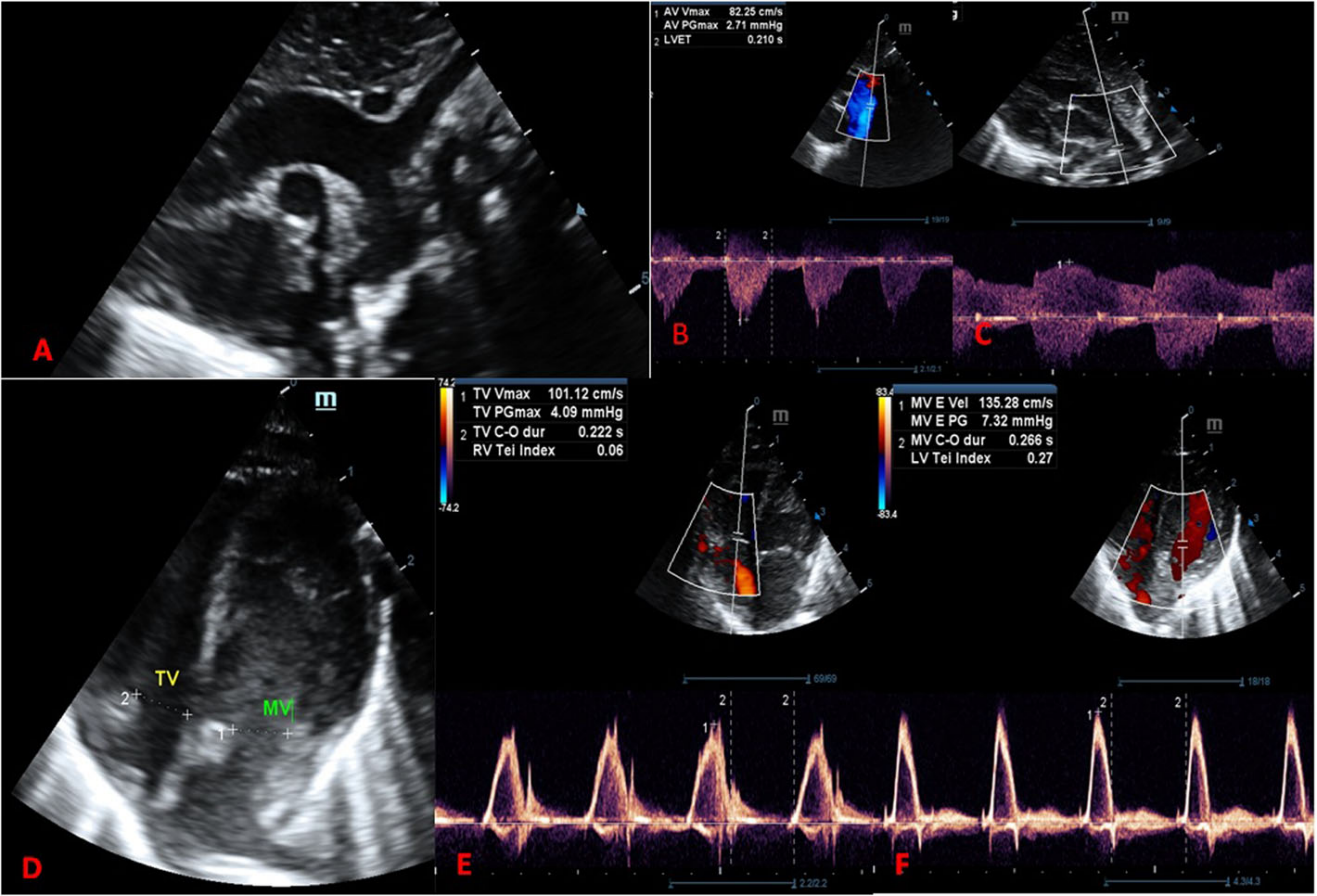
Echocardiography views at the age of 3 month: **A** Suprasternal view on 2D echo showing arch of aorta with variant of two arteries ascending from aortic arch. **B** Doppler parameters of blood flow in descending aorta. **C** Doppler blood flow of patent ductus arteriosus at the age of 3 months. **D** 4 chamber view with opened atroventricular valves. **E** monophasic tricuspid valve inflow. **F** monophasic mitral valve flow with increased velocity to 1,3 m/s. (written consent to publish was obtained from the patient’s parents)

The diagnosis of Turner syndrome prompted the diagnostics of other congenital abnormalities. Echocardiography did not show any significant structural defects (Fig. 7). The PDA was pharmacologically ligated with paracetamol. Ultrasound examinations of the abdomen did not show any abnormalities in the structure of the urinary system.

## Discussion

This article presents the clinical case of a preterm female infant diagnosed with haemophilia and Turner syndrome. First symptoms occurred on the second day after birth, manifesting as prolonged bleeding from injection sites. It was necessary for the medical team to adjust standard procedures to ensure effective monitoring, treatment and pre-immunisation screening.

Any preterm infant born at 28 weeks gestation is exposed to a number of invasive medical procedures: mechanical ventilation, central venous catheterisation, the collection of blood samples, patent ductus arteriosus ligation. Preterm babies are also at higher risk of central nervous system bleeding.

The risk of severe bleeding is particularly high in patients with neonatal haemophilia, which could pose a life threat, early diagnosis and commencement of treatment are vital for avoiding chronic health problems.

Haemophilia is a sex-linked recessive disorder. A sex-linked recessive disease is typically associated with one parent having the disease or the child being male. And there is a tendency to forget about other possible pathogenetic mechanisms underlying the disease, such as additional chromosomal aberrations, de novo mutations and skewed X-chromosome inactivation.

In the discussed clinical case the child’s father was healthy, while the mother’s brother had severe haemophilia. At first vitamin K deficiency, sepsis and DIC were determined as the initial differential diagnosis. Dual diagnosis of haemophilia and Turner syndrome was suspected only after repeating bleeding episodes from injection sites, marked dysmorphy and a thorough history taking.

Spontaneous bleeding is characteristic for haemophilia.

The disease can be severe, moderate, or mild. In the case of severe haemophilia, the first symptoms (bruises and haematomas) are correlated with the development of the child’s motor activity, i.e. typically when the child is around 2 years old [3]. This is when most cases are diagnosed.

Premature children display symptoms earlier - usually in the first days after birth [16–23]. This is connected with the significant number of invasive procedures they undergo at NICU.

Haemophilia symptoms in neonates are different. First symptoms and the most common are prolonged bleeding a few hours after disrupting the skin’s integrity, while collecting blood samples for laboratory tests [16–23], or signs of CNS bleeding. Moreover, in the neonatal period, one should be alarmed by repeated bleedings from wounds which appear to have closed, prolonged bleedings from wounds continuing for days or weeks, bleeding occurring a few days after a minor injury- e.g. a nodule on the head in presented case report, subcutaneous haematoma appearing several days after an injury, massive CNS bleeding, adrenal haemorrhage or subperiosteal haematoma.

The crucial element in the diagnostic and therapeutic activities in the neonatal period involves the regular monitoring of bleeding in the central nervous system, the abdominal cavity, the urinary tract, and the gastrointestinal tract, and observations for any potential signs of such bleeding (apnoea). These procedures enable the therapeutic team to immediately begin treatment to prevent permanent damage.

It should be noted that haemophilia alone is not a cause of CNS bleedings. It can only increase the risk of heavy CNS bleedings. No preterm infant with diagnosed haemophilia, according to PubMed database, experienced heavy CNS bleeding [7].

Preterm babies belong to the group of patients at risk of iatrogenic blood loss. The additional risk connected with spontaneous, prolonged minor bleeding predisposes them to excessive blood loss, and exposes to transfusions of packed red blood cells (Fig. 4).

Supplementation of the missing factor VIII is the basis of haemophilia A treatment [3]. Its main purpose is the prevention of spontaneous bleeding and obtain factor VIII activity above 1% [3].

Treatment of neonates and preterms is different. There is no preventive treatment. One study has pointed to the higher frequency of factor VIII inhibitor occurrence in patients with multiple exposures to factor VIII before the age of 2 [24].

Therefore the preterm girl received factor VIII only in the case of spontaneous prolonged bleeding, and every procedure connected with an increased risk of injury (central lines, surgical PDA closure, retinal laser photocoagulation, intramuscular injections, lumbar puncture, arterial blood gas, lung puncture, umbilical vessels catheterisation).

As with every medical substance, factor VIII therapy can also cause numerous adverse effects, usually allergic reactions (nausea, urticaria, rash, apnoea, cough, tight chest, wheezing, pressure drop, anaphylaxis) [3]. In addition, the transfer of infectious agents cannot be ruled out (Fig.3).

The most severe adverse effect of factor VIII treatment is appearance anti-factor VIII antibodies, what happened in 75 day of life of the premature girl.

Multiple risk factors conducive to the development of an inhibitor were present in the described girl: starting the administration of factor VIII at an early age, intensive treatment with factor VIII, an early infection, an ophthalmological procedure and the related presence of numerous pro-inflammatory signals, as well as recombinant factor VIII therapy [25, 26]. The results of a randomised study published this year, which covered previously untreated children, point to the fact that the inhibitor occurred more frequently in patients who received recombinant factors (28.5%) than in those who were given plasma-derived factors (13.8%) [27]. Moreover, the family history of inhibitor presence, an Afro-American origin, large deletions and nonsense mutations within the F8 gene, foster the creation of the inhibitor [26].

The appearance of these antibodies renders the substitutive administration of factor VIII ineffective.

The occurrence of an inhibitor forces the therapeutic team to modify the treatment of spontaneous bleeding, and to apply by-passing agents to obtain homeostasis. Such agents include concentrated products which activate blood clotting, and by-pass the stage dependent on the presence of factor VIII (recombinant activated factor VII (rFVIIa) and prothrombin complex concentrate (PCC), including activated prothrombin complex concentrate (APCC)) [3].

Inhibitors occur in up to 30% [25] of patients with severe haemophilia A, and are the most frequent in the first 50 days of treatment [26, 28].

Patients with diagnosed haemophilia (irrespective of the disease form and the presence of the factor VIII inhibitor) are subject to the same obligatory immunisation scheme as healthy people [5].

Vaccination is safe procedure, however there are special precautions. The treating physician is responsible for carrying out any obligatory vaccinations, and those recommended in accordance with the immunisation scheme, under the law of a given country [5].

The girl received subcutaneous injections instead of intramuscular ones, ice was applied on the injection site, and the smallest possible needles were used [5]. If any intramuscular vaccine are required, preventive factor VIII administration should be considered [5].

Vaccines do not affect the development of the factor VIII inhibitor [29], thus factor VIII substitution therapy and vaccination can be performed on the same day [5]. According to experts, delaying the immunisation by 24-72 h is not needed. Table 2 presents a summary of management with haemophilic preterm patient.

**Table 2:** Summary of management with haemophilic preterm patient

Labour and delivery	o Vaginal delivery or caesarean section o Minimalise risk of bleeding/haemorrhage- avoid prolonged labour o No vaccum o No forceps o No fetal scalp elecrodes o No fetal blood samping o Post-delivery screening of VIII clotting factor (umbilical cord) o No invasive procedures till results.	[3, 33]
Ventilation	o non-invasive and mechanical ventilation (all modes) if required o focus on nose care	[29, 34]
Central venous catheter	o factor replacement prior to procedure	[3]
Pawilizumab	o intramuscular injection o factor replacement prior to vaccination o application of ice to the injection site o compression at the injection site	[31]
Vaccination	o vaccinations (mandatory and recommended) according to institutional vaccination schedule o subcutaneous administration preferred o application of ice to the injection site o thinnest possible needle	[31]
Pain	o minimalisation of invasive procedures o cumulation of prcedures o no nonsteroidal anti-inflammatory drugs (NSAIDs) or acetylsalicylic acid (ASA) o usage of acetaminofen, morphine o tramadol, codeine, ketamine, fentanyl	[3]
Surgical procedures	o pre-operative inhibitor (anti- FVIII antibodies) screening o factor replacement therapy prior to procedure (or in case of presence of FVIII antibodies-therapy with by-passing coagulation agents) o reservation of VIII coagulation factor or by-passing agents for the surgery and postoperative time, rehabilitation o re-screen level of anti- FVIII antibodies after surgery o surgical procedures at or in consultation with a comprehensive hemophilia treatment centre.	[3]
PDA ligation	o usage of paracetamol, o avoid ibuprofen	[3, 29, 34]

A limitation in the presented case is the fact that haemophilia was diagnosed after birth. The mother did not have time to prepare for her child’s disease. It is mainly because most women do not know that they are haemophilia carriers [3].

It is important to note that the mother required the administration of packed red blood cells after her first childbirth. The mother is most likely a carrier of the haemophilia gene. She experienced discrete symptoms of bleeding diathesis without being aware of them. During pregnancy the concentration of factor VIII increases, and after childbirth it decreases, causing bleeding [3].

Women suspected of being carriers, with a positive family history, should be informed of the benefits of genetic testing before pregnancy. Such diagnostics will aid the appropriate planning of labour and early postnatal care [30].

Turner syndrome was also diagnosed postnatally, yet very early in the child’s life. Postnatal diagnostics are not always simple, particularly in the neonatal period or in preterm children when dysmorphia is not very evident. It can also be delayed until adulthood, when difficulties with getting pregnant point to such a diagnosis. Prolonged bleeding from injection sites expedited the diagnosis of Turner syndrome. However, it is also possible for such a patient to be discharged home without this diagnosis.

Karyoype evaluation revealed that the girl had a mosaic karyotype, with the presence of a marker chromosome (46,X + mar,45, X). Marker chromosome can be with or without significance for the patient, depending on which genes are affected. The marker chromosome can be an X or Y chromosome or an autosome fragment. Additional aneuploidies are quite frequent in Turner syndrome, and they can be vital for the further development of the child [4]. The most frequent aneuploidies are those affecting autosomes (such as trisomy 21) [4]. It is important to rule out the presence of the SRY gene (sex-determining region Y) which is responsible for the development of the male sex and increases the risk of gonadoblastoma in residual gonads [31–33].

## Conclusions

- Concomitant Turner syndrome and haemophilia is very rare
- Haemophilia diagnostics should always be considered in neonates with a tendency for prolonged bleeding
- In the case of coexisting atypical phenotypic features, further diagnostics for another genetic condition should be considered
- Children with haemophilia are subject to the obligatory immunisation scheme under the law of a given country.

## Data Availability

The datasets analyzed during the current study are available from the corresponding author on reasonable request.

## Abbreviations

APCC: Activated prothrombin complex concentrate
APTT: Activated partial thromboplastin time
CNS: Central nervous system
FFP: Fresh frozen plasma
INR: International normalized ratio
NICU: Neonatal Intensive Care Unit
PCC: Prothrombin complex concentrate
PDA: Patent ductus arteriosus
PT: Prothrombin time
RSV: Human respiratory syncytial virus
rFVIIa: recombinant factor VIIa
SRY: Sex-determining region Y
vWF: von Willebrand factor
